# Intrauterine fetal death due to rupture of umbilical vessels: a rare case of furcate cord insertion

**DOI:** 10.1186/s12884-024-06660-3

**Published:** 2024-07-05

**Authors:** Heng Xu, Jia-Ping Lu, Qiu-Lian Xu

**Affiliations:** Department of Obstetric, Jinhua People’s Hospital, Jinhua, China

**Keywords:** Umbilical vessels, Furcate insertion, Stillbirth, Prenatal examination

## Abstract

Furcate cord insertion refers to the separation of umbilical vessels before reaching the placenta, where the branching vessels normally attach at the edge of the placental parenchyma or near the placental membranes. This is an extremely rare abnormal umbilical cord insertion. This paper reported a case of a furcate cord insertion, where the rupture of exposed umbilical vessels led to intrauterine fetal death at full term. Through literature review, we analyzed the prenatal ultrasound characteristics and pregnancy outcomes of furcate cord insertions, with the aim to improve detection rates and reduce the risk of adverse pregnancy outcomes.

## Introduction

The umbilical cord is an important connecting structure between the fetus and the placenta. Under normal conditions, Wharton’s jelly encapsulates one umbilical vein and a pair of umbilical arteries, extending all the way to placental tissue. Furcate cord insertion is a condition where the umbilical vessels separate before reaching the placenta, with the branching vessels normally attaching at the edge of the placental parenchyma or near the placental membranes. Due to the loss of Wharton’s jelly protection at the separation site, umbilical vessels are highly prone to rupture and embolism, leading to bloody amniotic fluid and fetal death [1]. The incidence of furcate cord insertion is very low in only 0.1%, and research regarding furcate cord insertion is largely based on case reports [1]. Current prenatal ultrasound diagnoses of abnormal cord insertions mainly focus on velamentous insertion, but overlook the important feature of furcate insertion: the umbilical vessels freely enter the placental parenchyma [2, 3]. This paper reports a case of furcate cord insertion, in which the rupture of exposed umbilical vessels led to intrauterine fetal death. Through literature review, we analyze the prenatal ultrasound characteristics and pregnancy outcomes of furcate cord insertions, aiming to enhance understanding of this type of insertion abnormality in prenatal diagnostics, clinical management, and pregnancy outcomes.

## Case report

**Fig. 1:**
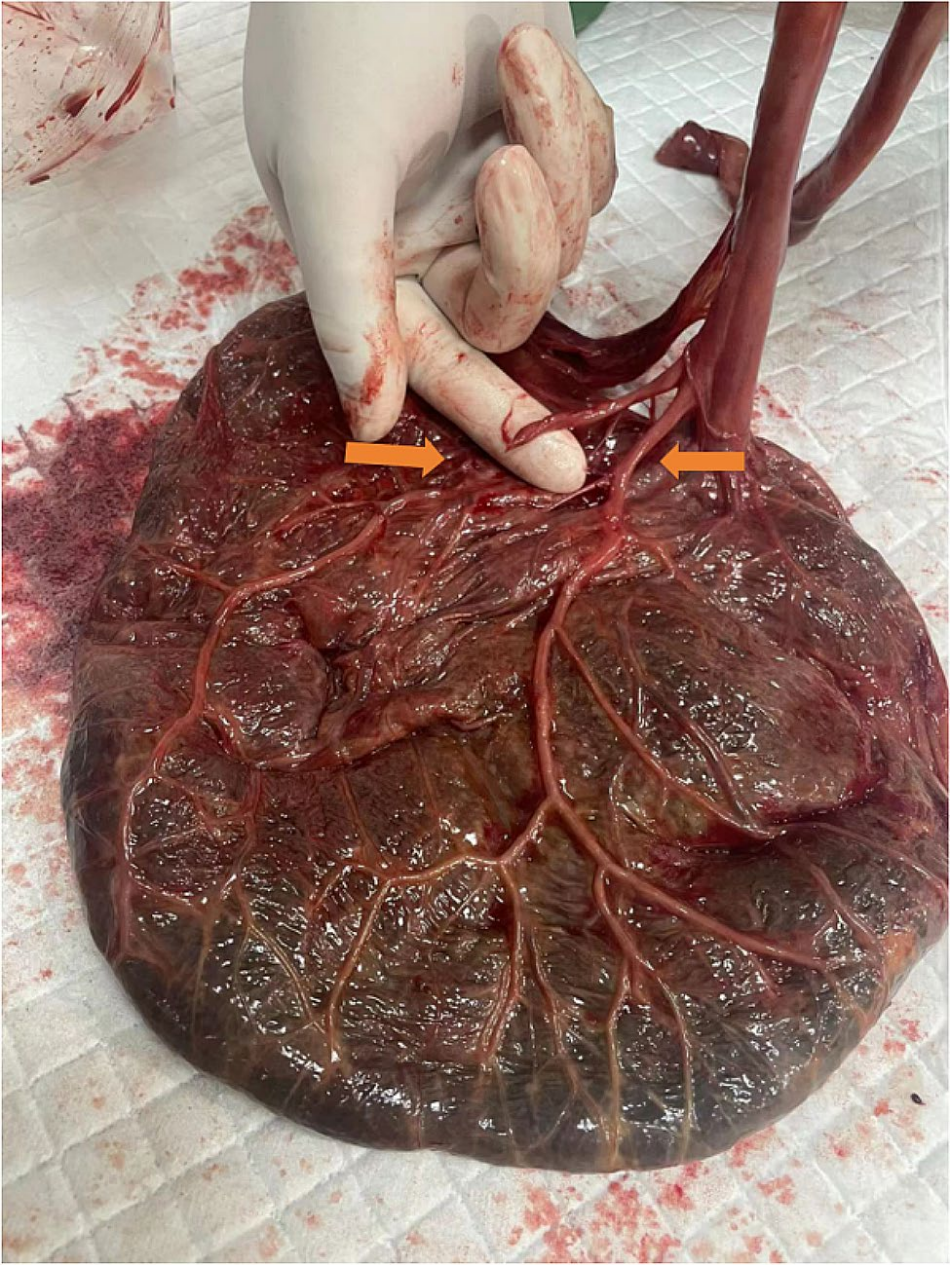
Macroscopic examination of placenta. The main umbilical vessels separated into several branches before reaching the placenta, and one of the free umbilical vessels was ruptured

Written informed consent for publication has been obtained from the participants in this study. The patient was a 30-year-old primigravida who was generally in good health and successfully passed all prenatal check-ups. When she was 40 weeks pregnant, she presented to the hospital due to a cessation of fetal movements for one day. Ultrasound examination revealed intrauterine fetal death. After admission, the patient was given rivanol for amniotic cavity injection to induce labor. During the amniotic cavity injection process, the amniotic fluid was found to be bloody upon retraction. To exclude the possibility of placental abruption, an ultrasound was repeated, which showed no signs of placental abruption. The patient vaginally delivered a male stillborn on the second day after induction of labor, weighing 3450 g. The neonate had a pale complexion, and the amniotic fluid was bloody with clots. The placenta was spontaneously delivered, with a furcate cord insertion. The main umbilical vessels separated into several branches before reaching the placenta, and one of the free umbilical vessels was ruptured (Fig. 1). The patient recovered well postpartum and was discharged two days after delivery.

## Discussion

### Mechanism of furcate cord insertion

The mechanism of furcate cord insertion is not yet clear. Normally, the extraembryonic mesoderm that connects the amniotic cavity and the trophoblastic cell layer (namely, the body stalk) forms the rudiment of the umbilical cord. As the amniotic cavity expands and the yolk sac evolves, the rudimentary umbilical cord and its internal yolk sac and urinary sac are compressed to form a rod-like structure. The amnion tightly covers its surface. The extraembryonic mesoderm that surrounds the umbilical vessels eventually evolves into a jelly-like tissue (i.e., Wharton’s jelly) to protect the umbilical cord from compression [4]. Some scholars believe that for some reason, the extraembryonic mesoderm connecting the amniotic cavity and the trophoblastic cell layer widens, and the extraembryonic mesoderm surrounding the umbilical vessels does not develop or subsequently degenerates, leading to the separation of the umbilical vessels. With the development of the placenta, the separation of the umbilical vessels becomes more apparent, forming a furcate insertion [1].

### Key points in ultrasonic diagnosis of furcate umbilical cord insertion

Prenatal reports diagnosing the furcate insertion of the umbilical cord are rare, with most anomalies in the umbilical cord discovered through ultrasound examination. Reviewing the limited available ultrasound images and literature, we summarized the following sonographic characteristics of furcate umbilical cord insertion: ①The main trunk of the umbilical cord separates prematurely before entering the placenta; ②The separated umbilical vessels can manifest as branches of the umbilical arteries, branches of the umbilical vein, or a mixture of both; ③Umbilical vessel branches could be normal, marginally attached to the placental parenchyma, or directly attached to the adjacent amniotic membrane; ④Umbilical vessel branches may spiral around each other, run parallel, or run independently. Reviewing the literature on umbilical cord insertion abnormalities, past research has focused on the relationship between the location of umbilical cord insertion and the amniotic membrane, simply categorizing umbilical cord insertion abnormalities as marginal insertion and membranous (velamentous) insertion, while overlooking the furcate insertion [5]. Prenatal ultrasonography has difficulty detecting the manifestation of umbilical vessels being free from the fetal membranes in furcate attachment, especially when all the branches of the umbilical vessels shown by ultrasonography are entering the placenta. Therefore, how to distinguish between furcate and membranous insertion prenatally is both a key and difficult point.

Some research metaphorically referred to the sonographic sign of membranous insertion as the “Mangrove Sign”, describing the relationship between the main trunk and multiple branches (more than three) of the umbilical vessels in membranous insertion [6]. However, a literature review found that multiple articles reported the sign of multiple vessels (more than three) in the umbilical vessel branches of furcate umbilical cord insertion [7, 8]. The number of umbilical vessel branches is not a reliable indicator for distinguishing between velamentous insertion and furcate insertion. In terms of umbilical vessel course, sail-like attached umbilical vessel branches are usually seen as fan-shaped separation after the umbilical cord main trunk enters the amniotic membrane, and the umbilical vessel branches go directly along the fetal membrane into the placental parenchyma [9]. In contrast, furcate insertion is characterized by umbilical vessel branches free in the amniotic cavity after the separation of the umbilical cord main trunk, which may proceed in a spiral, parallel, or independent manner, indicating that the course of umbilical vessel branches can be used to differentiate the two types of insertion abnormalities.

**Fig. 2:**
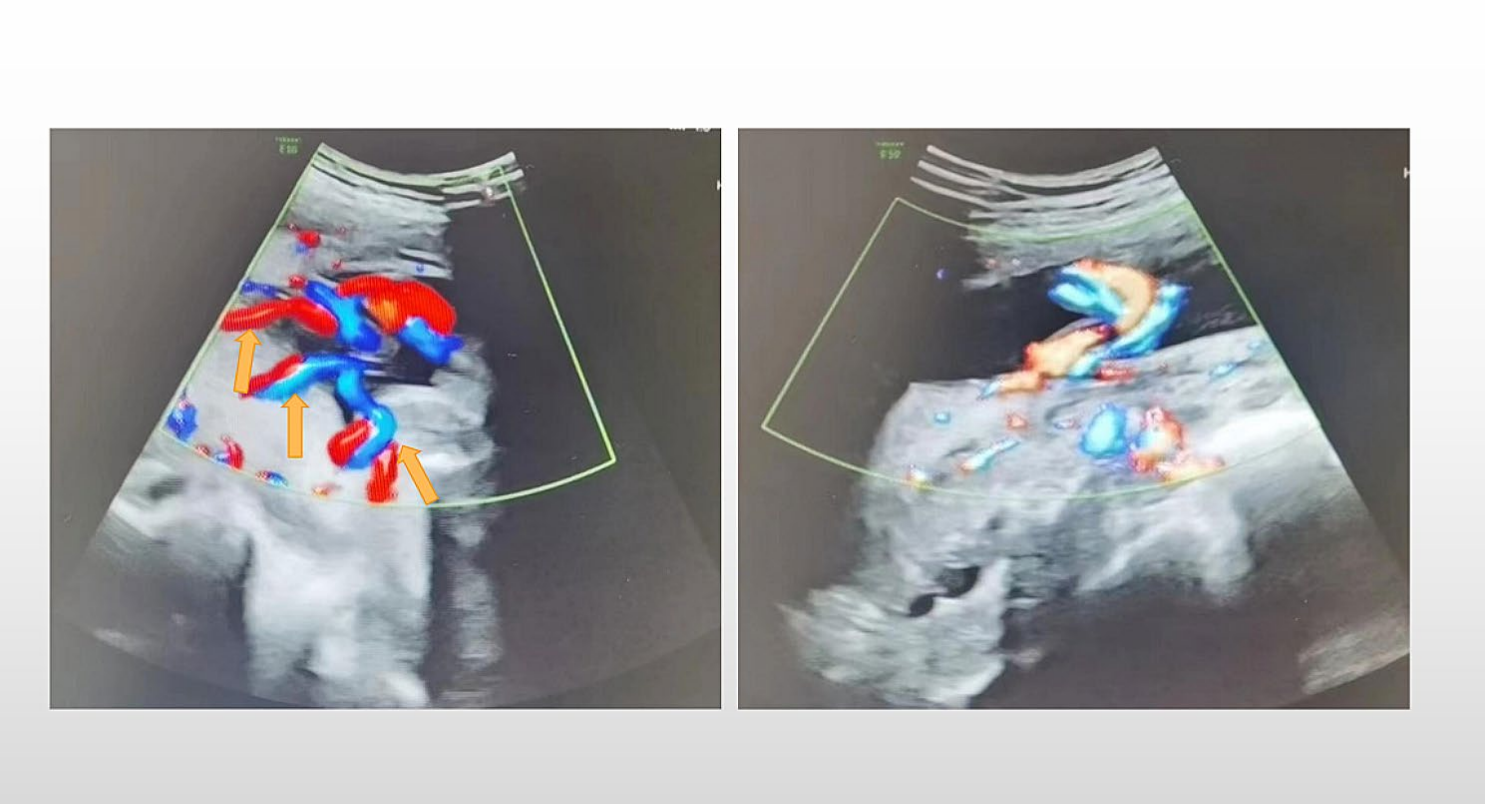
Ultrasonography Images. The vessels indicated by the yellow arrows may have been separated before entering the placenta

Upon retrospective review of this patient’s prenatal ultrasound images (Fig. 2), which suggested that the umbilical vessels may be separated before entering the placenta, and the umbilical vessels branch into the placental parenchyma. However, the sonographers at that time did not observe this abnormal appearance from more angles, which undoubtedly caused a difficult diagnosis.

### Pregnancy outcomes of furcate cord insertion

Although the branches of the umbilical vessels in furcate cord insertion enter the placental parenchyma, adverse pregnancy outcomes such as umbilical vessel dilation, rupture, thrombus formation, and intrauterine fetal death can occur due to premature separation of the main trunk of the umbilical cord in the amniotic cavity and the unprotected state of the separated umbilical vessel branches without Wharton’s jelly [2]. An article analyzing the clinical and pathological characteristics of 132 cases of furcate umbilical cord insertions found that the risk of intrauterine death caused by furcate insertion was 1.0%, with the main cause of death being umbilical vessel branch rupture, followed by umbilical vessel dilation or thrombus formation, and it was not significantly related to fetal growth restriction [1]. After reviewing the literature, we found there were only 12 case reports related to furcate cord insertion, of which only 5 reports made a prenatal diagnosis of furcate cord insertion [7, 8, 10–12]. These patients all chose induction of labor or cesarean section at 37–39 weeks, and the newborns did not show complications after birth. The remaining 7 case reports documented intrauterine fetal death due to spontaneous rupture of umbilical vessels [13–19].

In conclusion, furcate umbilical cord insertion is an abnormal insertion method that differs from membranous (velamentous) insertion and can increase the risk of adverse perinatal pregnancy outcomes. Due to the limited number of cases of furcate insertion, further large-scale studies are required to understand whether furcate insertion impacts the mode and gestational week of delivery. If the separation of the main trunk of the umbilical cord or its branching before entering the placental parenchyma is discovered during prenatal ultrasound examination, the possibility of furcate insertion should be considered. The course of the umbilical vessels should be closely tracked and monitored to minimize the risk of umbilical vessel dilation, rupture, thrombosis formation, or even intrauterine fetal demise.

## Data Availability

All data are available in the manuscript.
